# Construction and validation of a ferroptosis-related long noncoding RNA signature in clear cell renal cell carcinoma

**DOI:** 10.1186/s12935-022-02700-0

**Published:** 2022-09-14

**Authors:** Zhenpeng Zhu, Cuijian Zhang, Jinqin Qian, Ninghan Feng, Weijie Zhu, Yang Wang, Yanqing Gong, Xuesong Li, Jian Lin, Liqun Zhou

**Affiliations:** 1 Department of Urology, Peking University First Hospital, Beijing, 100034 China; 2 Institution of Urology, Peking University, Beijing, 100034 China; 3 National Urological Cancer Center, Beijing, 100034 China; 4 Beijing Key Laboratory of Urogenital Diseases (Male) Molecular Diagnosis and Treatment Center, Beijing, 100034 China; 5 The Affiliated Wuxi Second People’s Hospital of Nanjing Medical University, Wuxi, 214000 China

**Keywords:** Ferroptosis, Clear cell renal cell carcinoma, Molecular subtyping, Prognostic signature, Long Noncoding RNA

## Abstract

**Background:**

Clear cell renal cell carcinoma (ccRCC) is characterized by the accumulation of lipid-reactive oxygen species. Ferroptosis, due to the lipid peroxidation, has been reported to be strongly correlated with tumorigenesis and progression. However, the functions of the ferroptosis process in ccRCC remain unclear.

**Methods:**

After sample cleaning, data integration, and batch effect removal, we used the Cancer Genome Atlas (TCGA) and International Cancer Genome Consortium (ICGC) databases to screen out the expression and prognostic value of ferroptosis-related lncRNAs and then performed the molecular subtyping using the K-means method. Then, the functional pathway enrichment and immune microenvironment infiltration between the different clusters were carried out. The results showed a significant difference in immune cell infiltration between the two clusters and the associated marker responded to individualized differences in treatment. Then, least absolute shrinkage and selection operator (LASSO) Cox regression was used to establish a prognostic signature based on 5 lncRNAs. This signature could accurately predicted patient prognosis and served as an independent clinical risk factor. We then combined significant clinical parameters in multivariate Cox regression and the prognostic signature to construct a clinical predictive nomogram, which provides appropriate guidance for predicting the overall survival of ccRCC patients.

**Results:**

The prognostic differentially expressed ferroptosis-related LncRNAs (DEFRlncRNAs) were found, and 5 lncRNAs were finally used to establish the prognostic signature in the TCGA cohort, with subsequently validation in the internal and external cohorts. Moreover, we conducted the molecular subtyping and divided the patients in the TCGA cohort into two clusters showing differences in Hallmark pathways, immune infiltration, immune target expression, and drug therapies. Differences between clusters contributed to individualizing treatment. Furthermore, a nomogram was established to better predict the clinical outcomes of the ccRCC patients.

**Conclusions:**

Our study conducted molecular subtyping and established a novel predictive signature based on the ferroptosis-related lncRNAs, which contributed to the prognostic prediction and individualizing treatment of ccRCC patients.

**Supplementary Information:**

The online version contains supplementary material available at 10.1186/s12935-022-02700-0.

## Introduction

Renal cell carcinoma (RCC) is one of the most common malignant tumors of the urinary system [1]. As the major pathologic subtype of RCC, clear cell renal cell carcinoma (ccRCC), especially metastatic ccRCC, often has high morbidity and mortality [2]. Approximately 25–30% of ccRCC patients have metastasis at initial diagnosis, which usually indicates poor prognosis. Moreover, the tumor-node-metastasis (TNM) staging system, which is currently applied in clinical practice, is considered less accurate in evaluating the prognosis and progression of ccRCC patients [3]. Meanwhile, 73–75% of identified ccRCC driver aberrations were subclonal, which might contribute to different clinical outcomes [4, 5]. Hence, performing suitable molecular subtyping and exploring new prognostic signatures to diagnose and evaluate the prognosis of ccRCC patients remains significant.

During the past decades, ferroptosis has been gradually identified as an iron-dependent, nonapoptotic cell death mode characterized by the accumulation of lipid reactive oxygen species [6, 7]. An increasing number of studies have shown that dysregulation of ferroptosis-related genes (FRGs) plays an important role in the occurrence and development of many diseases, especially cancer [8–10]. Studies related to ferroptosis have become the focus in treating and detecting related diseases. Moreover, as the noncoding RNAs play an emerging role in cancer, long noncoding RNAs (lncRNAs) have been fully studied [11]. Interestingly, an increasing number of studies have demonstrated that lncRNAs can participate in the ferroptosis process and then influence tumor development [12–14]. Therefore, exploring ferroptosis-associated lncRNAs might provide new ideas and insights for ccRCC treatment and prediction.

In this study, we first explored the potential biological functions and correlation of FRGs. Afterward, we screened the prognostic ferroptosis-related lncRNAs (FRlncRNAs) and conducted the molecular subtyping of ccRCC patients. The correlation between the molecular clusters and immune cell infiltration was explored. Afterward, we conducted the LASSO Cox regression to establish a 5-ferroptosis-related LncRNAs signature in ccRCC patients and validated it in the ICGC database. Furthermore, a nomogram was constructed integrating the prognostic signature and significant clinical parameters. The results showed that a good predictive performance for the overall survival (OS) of ccRCC patients.

## Materials and methods

### Data selection and processing

The ccRCC sequencing data (HTSeq-FPKM) and the latest corresponding clinical information (Additional file 1: Table S1) were downloaded from the TCGA database (https://cancergenome.nih.gov/), including 539 ccRCC samples and 72 normal controls. Moreover, we downloaded the transcriptome profile and corresponding survival data from the ICGC database (http://daco.icgc.org/) as the validation cohort, including 91 RCC samples. Then, we distinguished between lncRNAs and mRNAs using the human GTF annotation file. The Matrix processing and batch effect removal were conducted via the *limma* and *sva* packages in R (v 4.0.3).

### Cell lines and clinical specimens

Seven kidney and RCC cell lines (kidney cell lines: HK-2; RCC cell lines: 786-O, 769-P, OS-RC-2, A498, ACHN, and Caki-1) were obtained from the American Type Culture Collection (ATCC). All cells were maintained in RPMI-1640 (Corning, USA) or high-glucose DMEM medium (Gibco, USA) with 10% fetal bovine serum (BI, Israel) and 1% penicillin and streptomycin (Gibco, USA) at 37 °C and 5% CO_2_. Ten paired ccRCC and adjacent normal tissues were obtained from ccRCC patients undergoing surgical resection at Peking University First Hospital. Detailed information on the 10 paired tissue specimens is shown in Additional file 2: Table S2. The Ethics Committee approved this study of PUFH, and all patients signed informed consent forms. All procedures were performed according to the World Medical Association Declaration of Helsinki.

### Real-time quantitative PCR (qPCR)

Total RNA of 10 paired clinical samples and 7 cell lines were extracted by Takara kit according to the manufacturer's protocol. Then, the RNA was reverse transcribed to cDNA in a 20 µl reaction system. All gene transcripts were quantified by qPCR using SYBR Premix ExTaq kit, and TUBA was used as a normalization control. The primer sequences are listed in the Additional file 3: Table S3. Each reaction was performed four times, and the 2^^−△△CT^ method was used to calculate the relative mRNA expression level.

### Identification of prognostic ferroptosis-related differentially expressed lncRNAs

According to previous studies [15–17], we obtained 259 FRGs, and the list is shown in Additional file 4: Table S4. After that, we screened ferroptosis-related lncRNAs with a filter (correlation  > 0.5, p < 0.01), including 2854 lncRNAs. The *limma* package was used to determine the differentially expressed lncRNAs (DElncRNAs) between the ccRCC patients and normal controls, including 1333 lncRNAs [18]. We intersected the DELncRNAs and ferroptosis-related LncRNAs to obtain the ferroptosis-related DElncRNAs (FRDElncRNAs). Univariate Cox regression of OS was performed on the FRDElncRNAs. Those with p < 0.01 were considered prognostic FRDElncRNAs.

### Molecular subtyping in ccRCC based on prognostic FRDElncRNAs

After obtaining prognostic FRDELncRNAs, we performed consensus clustering to identify the molecular subtypes of ccRCC by using the *ConsensusClusterplus* R package [19]. We selected 80% of the prognostic FRDElncRNAs resampling 100 times and determined clusterings of specified cluster counts (k). After this, the pairwise consensus values were calculated and stored in a symmetrical consensus matrix for each k. The k, at which there was no appreciable increase, was determined by the cumulative distribution function (CDF) plot and delta area plot. The alteration in immune infiltration between different clusters was estimated using the CIBERSORT method (Additional file 5: Figure S1).

### Potential biological functional enrichment

To gain insights into the cellular functions directly regulated by FRG transcriptional control, we compared the list of FRGs to the biological pathways annotated by the Kyoto Encyclopedia of Genes and Genomes (KEGG) [20]. Afterward, according to the two clusters, Gene Set Variation Analysis (GSVA) was conducted using the GSVA package in R software v.4.0.3 to investigate the enrichment of HALLMARK pathways with the h.all.v7.4.symbols.gmt gene set from the Molecular Signature Database [21].

### Analysis of potential therapeutic targets in different clusters

According to the two divided clusters, we determined whether there were differences in treatment effects between groups based on relevant databases. Since targeted drugs are commonly used to treat advanced kidney cancer, we used the R *pRRophetic* package to estimate drug response as determined by the half-maximal inhibitory concentration (IC50) for each kidney cancer patient on the Genomics of Drug Sensitivity in Cancer (GDSC) website [22]. The drug predictive model was applied to the processed, standardized, and filtered clinical tumor expression data, and then it estimated the drug sensitivity for each patients. Furthermore, based on the Cancer Immunome Altas (TCIA) database (http://tcia.at/), the Immunophenoscore (IPS) was obtained [23, 24]. To predict sensitivity to immunotherapy between different clusters, we compared the IPS of the two clusters in different immunotherapy decisions.

### Construction of the prognostic predictive risk signature

First, the TCGA cohort patients were randomly divided a the training set and an internal validation set. Meanwhile, the patients in the ICGC cohort were used as the external validation cohort. Based on the prognostic FRDELncRNAs, we performed LASSO Cox regression using the *glmnet* R package. We calculated each patient's risk score using the regression coefficient score of the individual lncRNAs and their expression values. We defined the formula for calculating the prognostic risk score as follows: Risk score = coef(Lnc1)*Exp(Lnc1) + coef(Lnc2)* Exp (Lnc2) + … + coef(Lncn)* Exp (Lncn), where “coef” represents the coefficient score estimated by LASSO Cox regression, and “Exp” represents the expression value of the individual LncRNAs. The detailed information of the signature is shown (Table 1). Then, we classified the ccRCC patients into the high- and low-risk groups, according to the median risk score of the training group as the cutoff [25].

**Table 1:** Detailed information of the LncRNAs in the prognostic signature

Gene	Ensembl ID	Description	Located	Coef.
LINC00460	ENSG00000233532	Long intergenic non-protein coding RNA 460	13q33.2	0.05808
LINC00894	ENSG00000235703	EOLA2 divergent transcript	Xq28	0.08831
VPS9D1-AS1	ENSG00000261373	VPS9D1 antisense RNA 1	16q24.3	0.11775
CYTOR	ENSG00000222041	Cytoskeleton regulator RNA	3q13.2	0.01316
FOXD2-AS1	ENSG00000237424	FOXD2 adjacent opposite strand RNA 1	1p33	0.07545

### Validation of the prognostic risk signature

We conducted the Kaplan–Meier and receiver operating characteristic (ROC) curve analyses to assess the prognostic risk signature’s validity. According to the calculated median risk score, all samples in each group were divided into high- and low-risk groups, and the *survminer* and *timeROC* packages were used to validate the predictive accuracy in the training and validation sets. The area under the curve (AUC) values corresponding to 1-, 3-, and 5-years were calculated. The time-dependent ROC curve was used to validate the predictive performance of the signature. An AUC value of 0.75 or higher was considered the significant predictive value, and the value of 0.60 or higher was regarded as acceptable for prediction. Furthermore, univariate and multivariate Cox regression was conducted to explore whether the ferroptosis-signature (FerroSig) could serve as an independent factor.

### Construction and validation of the nomogram

To better predict the prognosis of patients with ccRCC, we established a predictive nomogram based on clinical parameters and prognostic signature [26]. In brief, we first performed univariate and multivariate Cox regression analyses to identify clinical parameters and risk scores that could be used as independent risk factors. Subsequently, the significant factors were used to construct the predictive nomogram. We then evaluated the nomogram effect using calibration curves and time-dependent ROC curves. An AUC value of 0.75 or higher was considered a significant predictive value, and a value of 0.60 or higher was regarded as acceptable for prediction.

## Results

### Identification of the prognostic FRDElncRNAs

**Fig. 1:**
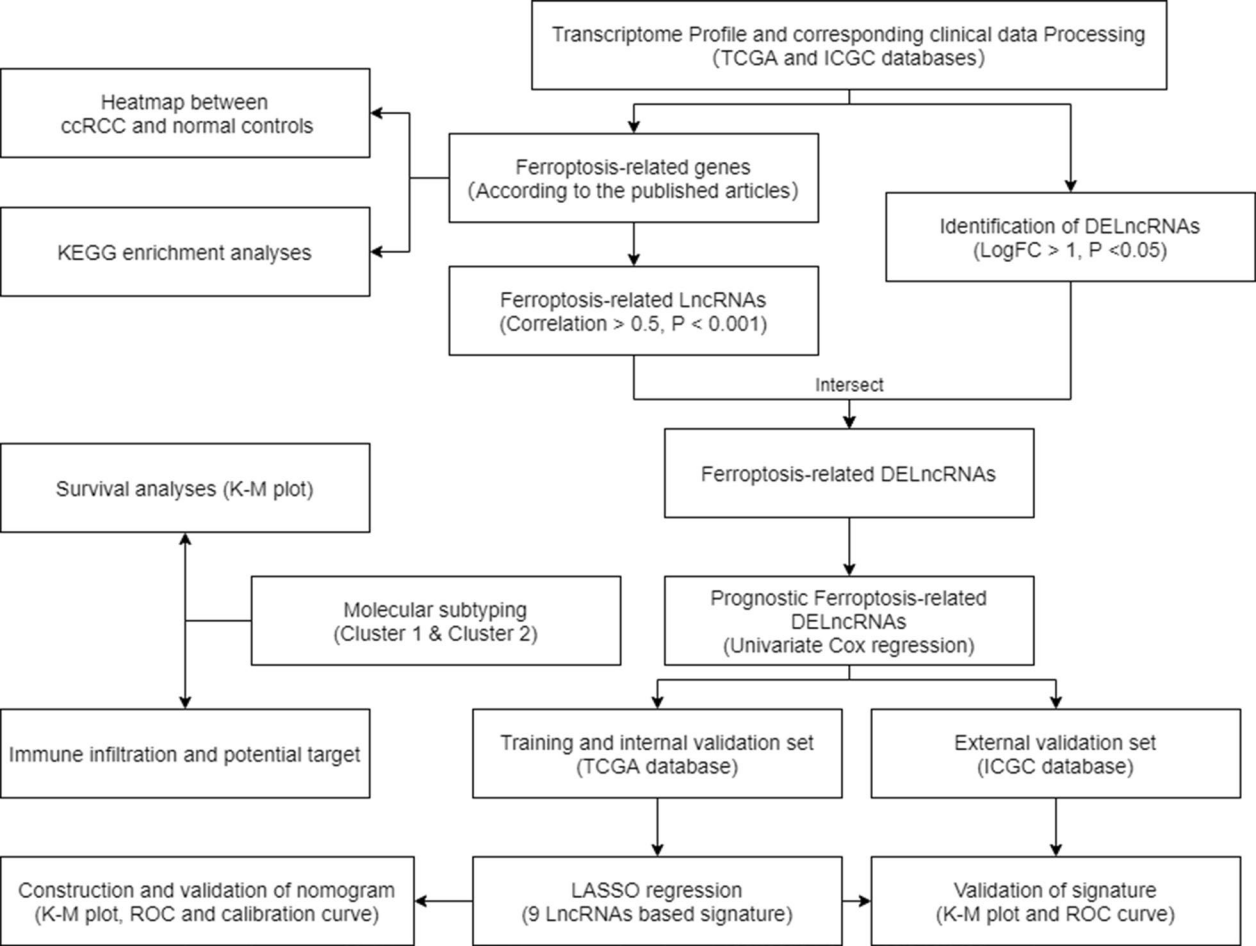
Flow chart of the whole analysis processes of this study

**Fig. 2:**
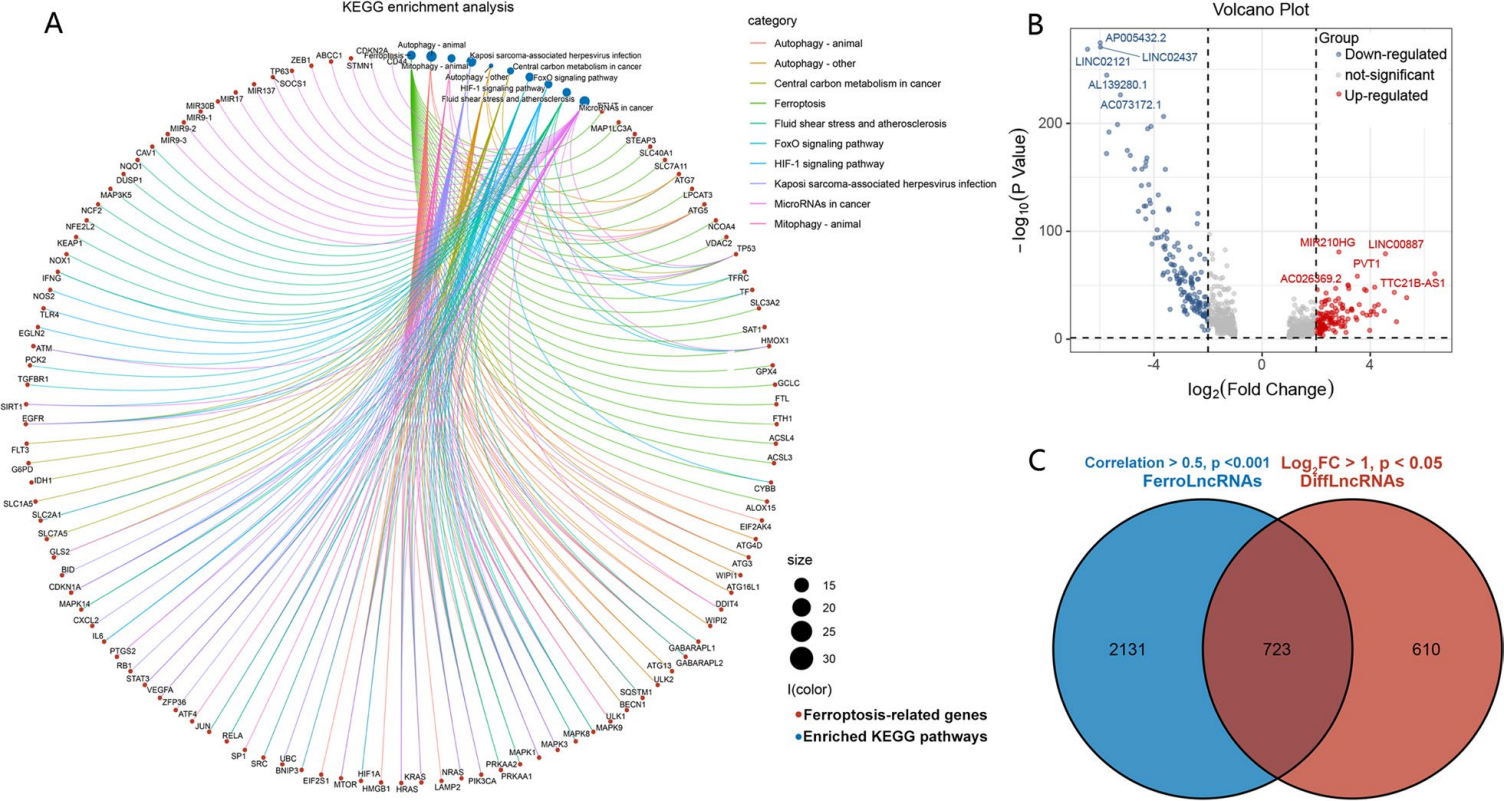
Function annotations of ferroptosis-related genes and Identification of the ferroptosis-related LncRNAs. **A** KEGG enrichment analysis of the ferroptosis-related genes, the larger the shape of the dot, the more corresponding genes are represented. **B** Volcano plot of the differentially expressed ferroptosis-related LncRNAs between the ccRCC and normal samples. The 5 most significant up-and down-regulated LncRNAs were labeled separately. **C** The Venn plot of the overlapped LncRNAs between two cohorts

The data processing was performed as described in the methods above. The flow chart of the whole process is shown (Fig. 1). To explore the potential functions of the FRGs, we first conducted KEGG pathway enrichment analysis. The results showed that FRGs were mainly enriched in ferroptosis and autophagy pathways (Fig. 2A). Afterward, we explored the FRlncRNAs with a correlation  > 0.5 and p < 0.01, using the *limma* package. Next, 1333 DElncRNAs between the ccRCC and normal samples from the TCGA set were screened, and the 5 most obvious upregulated- and downregulated lncRNAs were identified (Fig. 2B). Theoverlapping lncRNAs in both DElncRNAs and FRlncRNAs were identified as the FRDElncRNAs. There were 723 lncRNAs for OS selected as prognostic FRDELncRNAs for subsequent analyses (Fig. 2C).

### Molecular subtyping showed differences in therapeutic choice and immune microenvironments

**Fig. 3:**
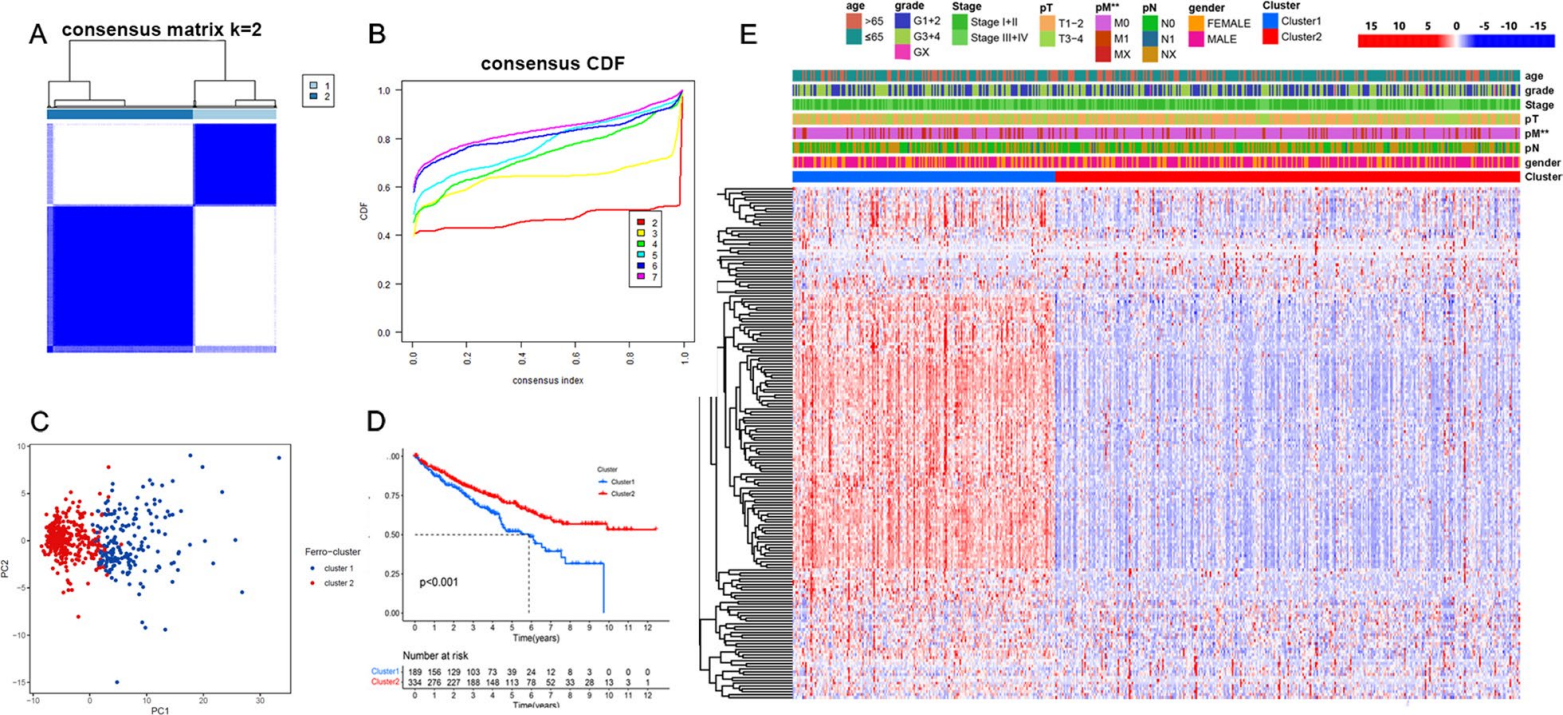
Molecular subtyping of the ccRCC patients based on the prognostic ferroptosis-related LncRNAs. **A** The heatmap corresponding to the consensus matrix for k = 2 was obtained by applying consensus clustering. Color gradients represent consensus values from 0–1; white corresponds to 0 and dark blue to 1. **B** Consensus among clusters for each category number k. **C** Principal Component Analysis and **D** K-M survival analysis of the two clusters. **E** The composite heatmap corresponding to the cluster and mRNA expression, TNM stage, AJCC stage, and ISUP grade, and Age as the annotations. **P < 0.01

The prognostic FRDElncRNAs above were used to screen the molecular subtypes of ccRCC by using the *ConsensusClusterPlus* packages. The K-means method was performed for clustering, and 80% of the lncRNAs were sampled 100 times using the resampling method. The consensus CDF and delta area were calculated to determine the clustering outcomes, as shown in Fig. 3B. When the cluster number was 2, there was no significant increase in the area under the CDF curve. Hence, we finally divided the samples in the TCGA cohort into cluster 1 and cluster 2. The representative consensus matrix of two clusters (Fig. 3A) displayed a well-defined 2-block structure. Principal component analysis (PCA) showed that the samples from two clusters could be well separated (Fig. 3C). Then, a heatmap integrating the expression of the prognostic FRDElncRNAs and clinical parameters in each subtype was generated, as is shown in Fig. 3D. The results showed that pM stage was higher in cluster1 than in cluster 2, which also clarified that the patients in cluster 1 had a worse prognosis.

### Comprehensive analyses of the molecular clusters

**Fig. 4:**
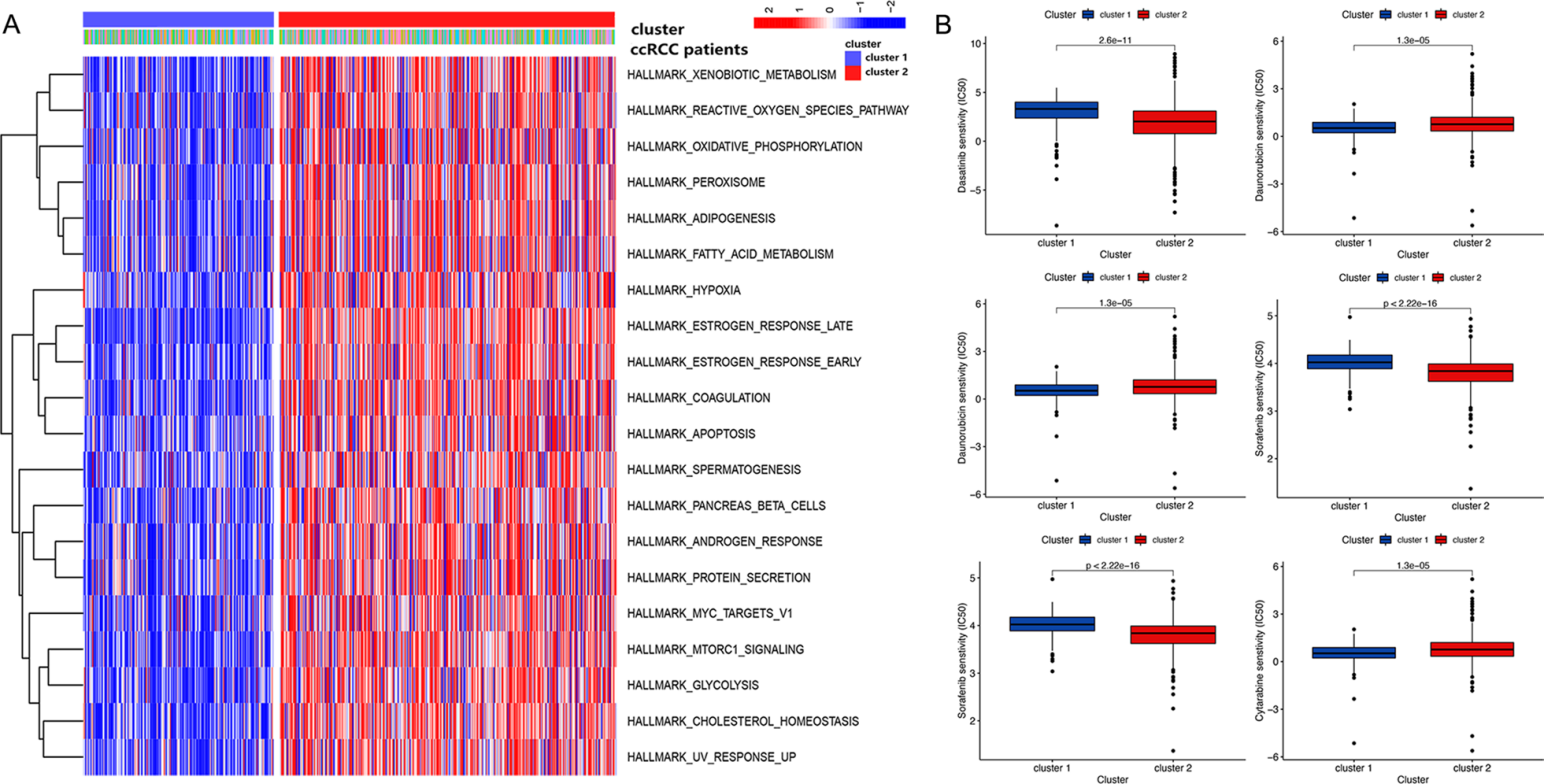
Biological functions and immune infiltration between two clusters. **A** Gene set variation analysis (GSVA) was performed to compute HALLMARK pathways between two clusters. **B** The IC50 data on drugs for ccRCC differentially expressed in the two clusters obtained by applying pRRophetic were shown

Subsequently, we explored HALLMARK pathway enrichments alterations between the two clusters using the GSVA method. The results showed that several oncogenic pathways were significantly altered between the two clusters, such as hypoxia and apoptosis (Fig. 4A). Furthermore, using the *pRRophetic* packages, we calculated and analyzed the IC50 between the two clusters for clinical drugs used for advanced ccRCC patients. According to the median IC50 value, the outcome indicated that the patients in cluster 2 were more sensitive to Daunorubicin and Tipifarnib. In contrast, using the Dasatinib, Paclitaxel, Sorafenib, and Pazopanib would be more effective for patients in cluster 1 (Fig. 4B).

**Fig. 5:**
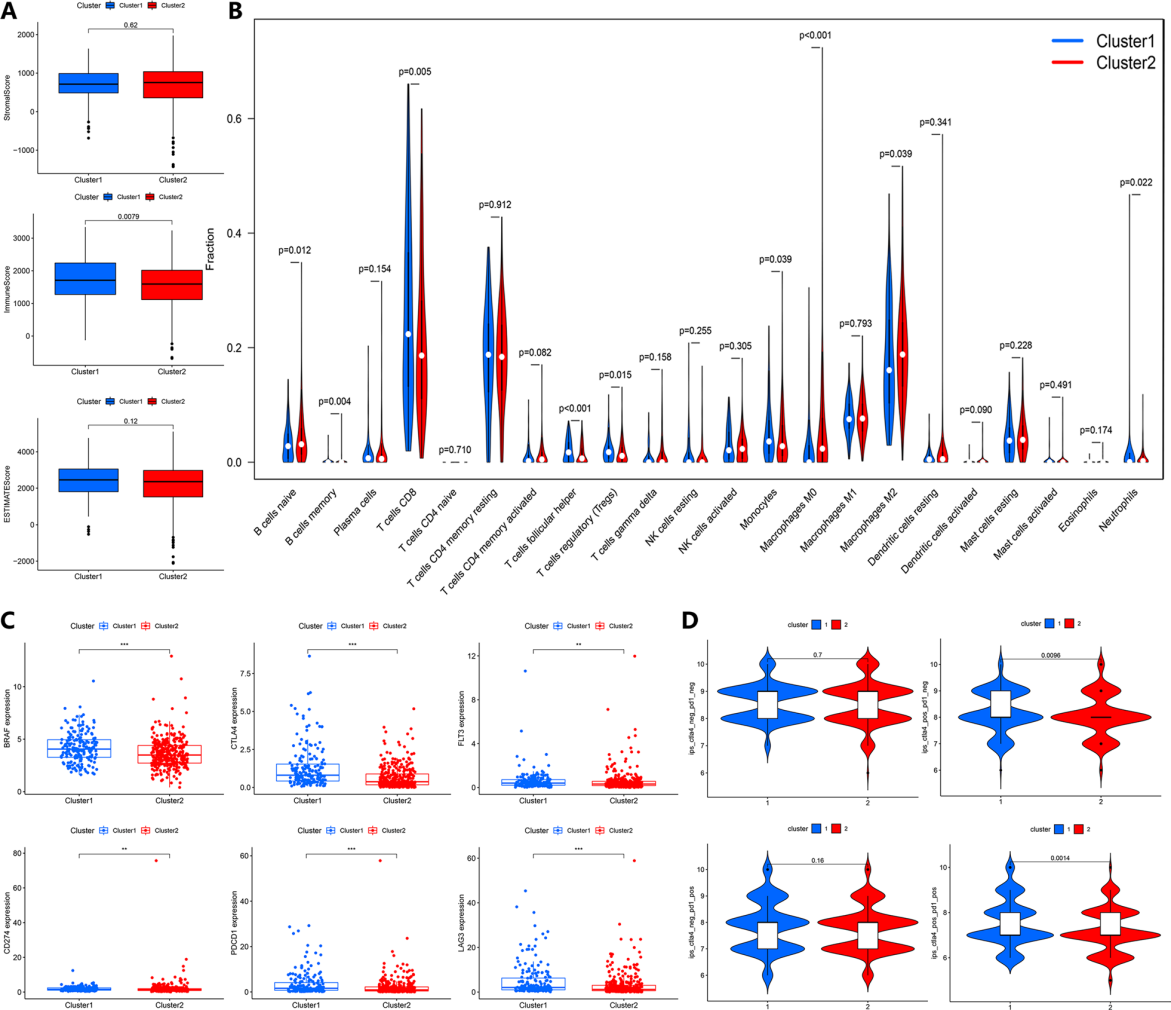
Tumor environment especially immune infiltration and potential immune therapeutic target between two clusters. **A** Tumor environment scores between two clusters. **B** The differentially expressed immune infiltrated cells in the two clusters obtained by applying Cibersoft were shown. **C** Common potential immune therapeutic targets between two clusters were shown. *P < 0.05, **P < 0.01, *** P < 0.001 **D** The different expressed of four immune status targets between two clusters were explored from the TCIA database

Since the tumor microenvironment (TME) plays a vital role in the development of tumors, we further explored the correlation between the TME and molecular clusters. As shown in Fig. 5A, we first calculated the stromal score, immune score, and ESTIMATE score. Only the immune score showed a significant difference between the two clusters. Hence, we then explored the immune cell infiltration between two clusters using the CIBERSORT method. Cluster 1 exhibited a higher infiltration of CD8 T cells, regulatory CD4 T cells, and Neutrophils. In comparison, cluster 2 showed a higher infiltration of macrophages (Fig. 5B). Meanwhile, the expression levels of some potential immunotherapy targets changed significantly in both classifications. The mRNA expression levels of potential immune therapeutic targets, such as BRAF and PD-1, were markedly higher in cluster 1 (Fig. 5C). Next, we explored the TCIA database for differences in the presence of immunotherapeutic targets. Previous studies reported the role of IPS in predicting the immunotherapy response of ccRCC patients. According to the IPS, the outcome showed that patients in Cluster 1 obtained a higher IPS of CTLA4 and CTLA4 + PD-1 than Cluster 2 (Fig. 5D). These results suggest that the cluster 1 group may be more sensitive to immunotherapy.

### Construction and validation of the prognostic signatures based on the prognostic FRDELncRNAs

**Fig. 6:**
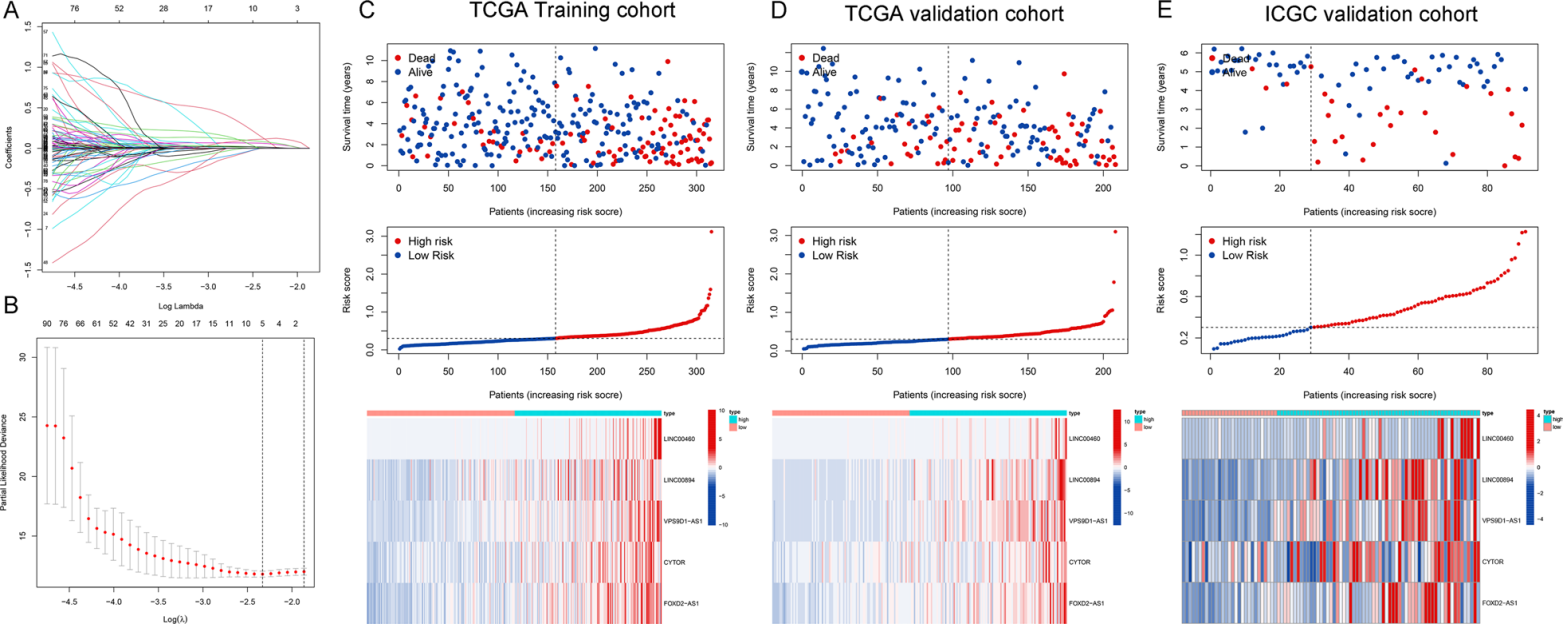
Establishment of the 5-LncRNAs based prognostic signature. **A** LASSO coefficient profiles of the prognostic DEFRGs. **B** Partial likelihood deviance was plotted versus log (Lambda). The vertical dotted line indicates the lambda value with the minimum error and the largest lambda value. (C-E) LncRNA expression patterns and the distribution of survival status increased risk score in the TCGA training set, TCGA internal validation set, and ICGC external validation set

**Fig. 7:**
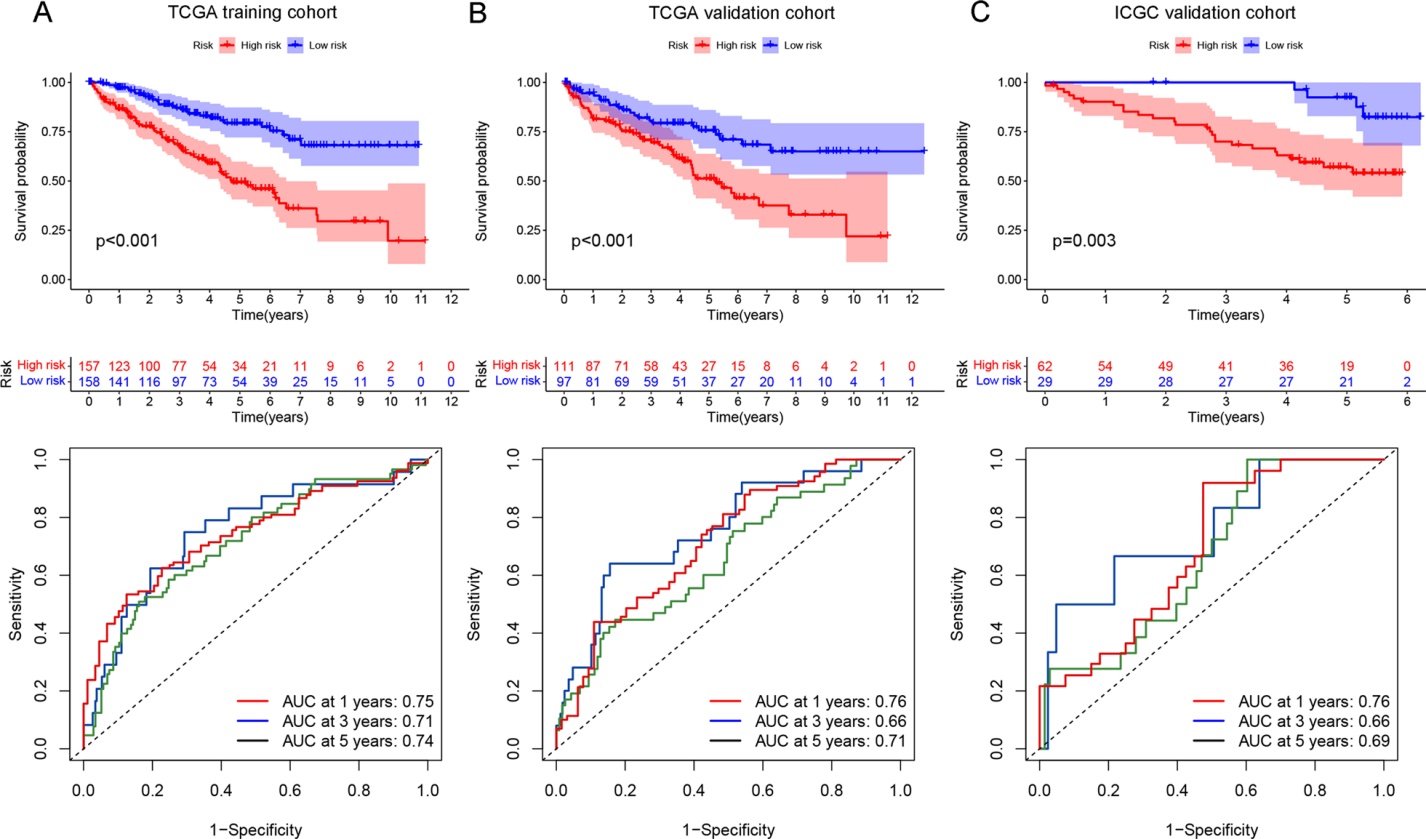
Validation of the prognostic signature. K-M plot analyses between the high-and low-risk patients in the TCGA training cohort **A**, TCGA validation cohort **B**, and ICGC validation cohort **C**. The 1-, 3-, 5-year time-dependent ROC curves in the TCGA training cohort (**A**), TCGA validation cohort (**B**), and ICGC validation cohort (**C**)

Based on the prognostic FRDElncRNAs obtained from the univariate Cox regression analysis, we constructed a 5 FRDElLncRNAs based prognostic signature using the LASSO regression (Fig. 6A and B). Detailed information on the lncRNAs from the signature is shown in Table 1. The risk score was constructed according to the following formula: risk score = 0.058 (LINC00460) + 0.088 (LINC00894) + 0.118 (VPS9D1-AS1) + 0.013 (CYTOR) + 0.075 (FOXD2-AS1).  Then, we explored the prognosis of TCGA-KIRC patients affected by the expression levels of the five LncRNAs, including overall survival and disease-free survival (Additional file 5: Fig. S1). Then, based on the calculated median risk score cutoff, patients were divided into the high- and low-risk groups. The risk score distribution, survival status, and expression of ncRNAs from the signatures are exhibited in the TCGA training cohort, TCGA validation set, and ICGC validation set are exhibited in Fig. 6C–E. The Kaplan–Meier log-rank test and the time-dependent ROC curve were used to evaluate the predictive ability and accuracy of the prognostic signature. The results of the Kaplan–Meier log-rank test showed that the high-risk group had a significantly worse OS than the low-risk group in the TCGA training set (Fig. 7A), TCGA validation set (Fig. 7B), and ICGC validation set (Fig. 7C). Moreover, the time-dependent ROC curve proved the 1-year, 3-year, and 5-year predictive accuracy of the signature for OS (Fig. 7A–C).

### Construction and validation of the prognostic nomogram

After establishing and validating the signatures based on the prognostic FRDElncRNAs, univariate and multivariate Cox regression analyses were used to explore independent risk factors in the TCGA dataset. As shown in Table 2, univariate Cox regression analysis showed that AJCC stage, ISUP grade, age, and the risk signature were significantly correlated with OS (Table 2). Moreover, the multivariate Cox regression analyses of the clinical parameters above showed that AJCC stage, ISUP grade, age, and the risk signature, were significantly correlated with OS (Table 2).

**Table 2:** Univariate and multivariate Cox analyses of clinical parameters and risk signature

Parameters	Univariate analysis		Multivariate analysis	
	HR (95%CI)	P value	HR (95%CI)	P value
Gender	0.963 (0.703, 1.319)	0.815	0.964 (0.700, 1.326)	0.820
AJCC stage	1.870 (1.638, 2.136)	< 0.001	1.597 (1.373, 1.859)	< 0.001
ISUP grade	2.251 (1.835, 2.763)	< 0.001	1.326 (1.051, 1.673)	0.017
Age	1.690 (1.241, 2.303)	< 0.001	1.567 (1.145, 2.145)	0.005
RiskSig	6.535 (4.608, 9.267)	< 0.001	3.733 (2.513, 5.546)	< 0.001

**Fig. 8:**
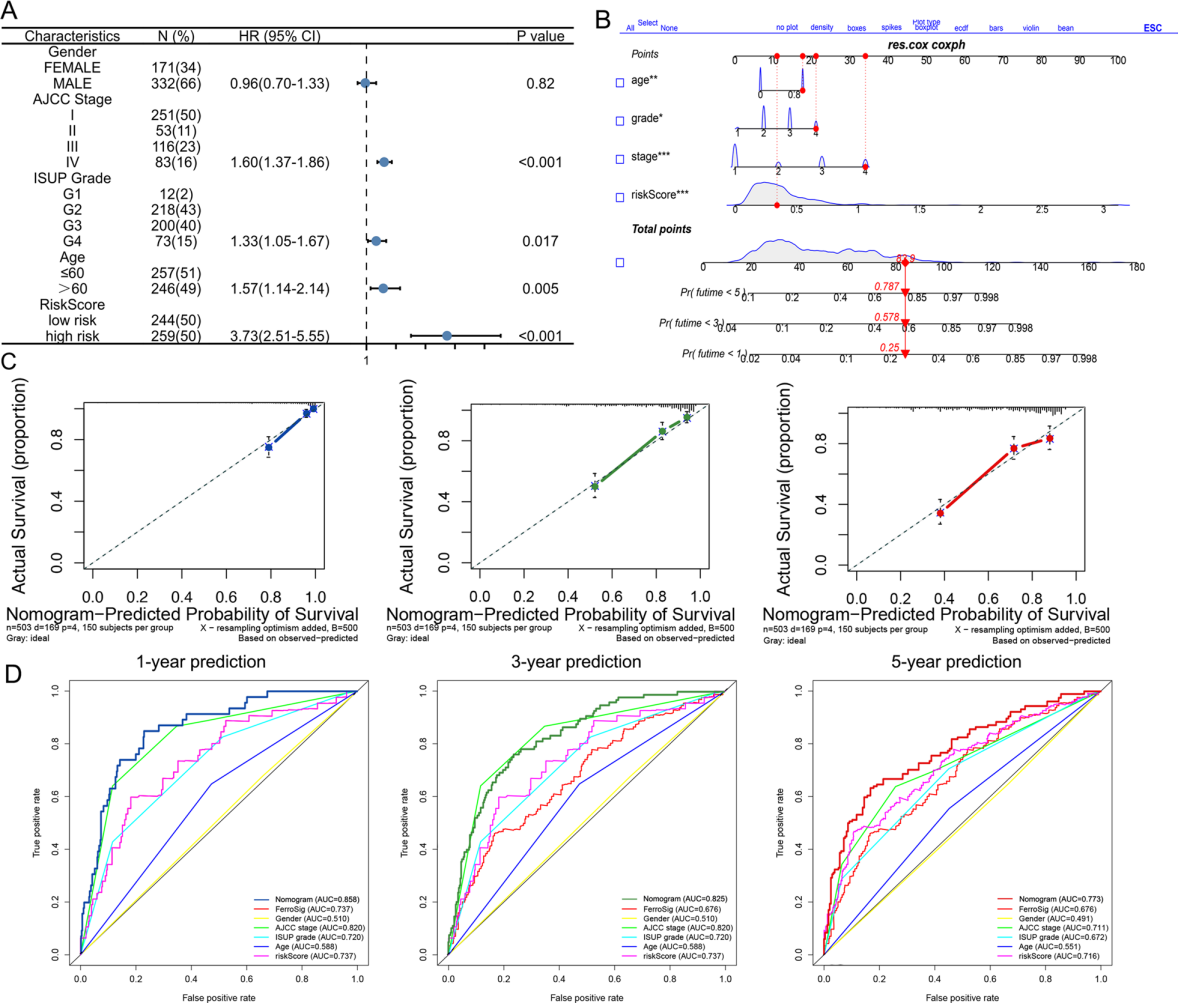
Construction and validation of the prognostic nomogram. **A** Forest plot of the significant clinical parameters in the multivariate Cox regression. **B** The nomogram based on the significant clinical parameters and risk signature **C** Calibration curves of the nomogram for 1-, 3-, and 5-year survival prediction. **D** The predictive value of the nomogram, risk signature, and clinical parameters

To better assess patient prognosis and guide clinical decision-making, we established a nomogram integrating the risk signature and significant clinical parameters in the multivariate Cox regression analyses (Fig. 8A). The C-index of 0.773 showed a good agreement, and the established nomogram was shown (Fig. 8B). The calibration curves showed that the predictive nomogram could well predict the survival status of patients at 1, 3, and 5 years (Fig. 8C). Additionally, the nomogram showed better predictive value than clinical indicators and the FRlncRNAs signatures (Fig. 8D).

### Validation of the lncRNAs in cell lines and clinical specimens

**Fig. 9:**
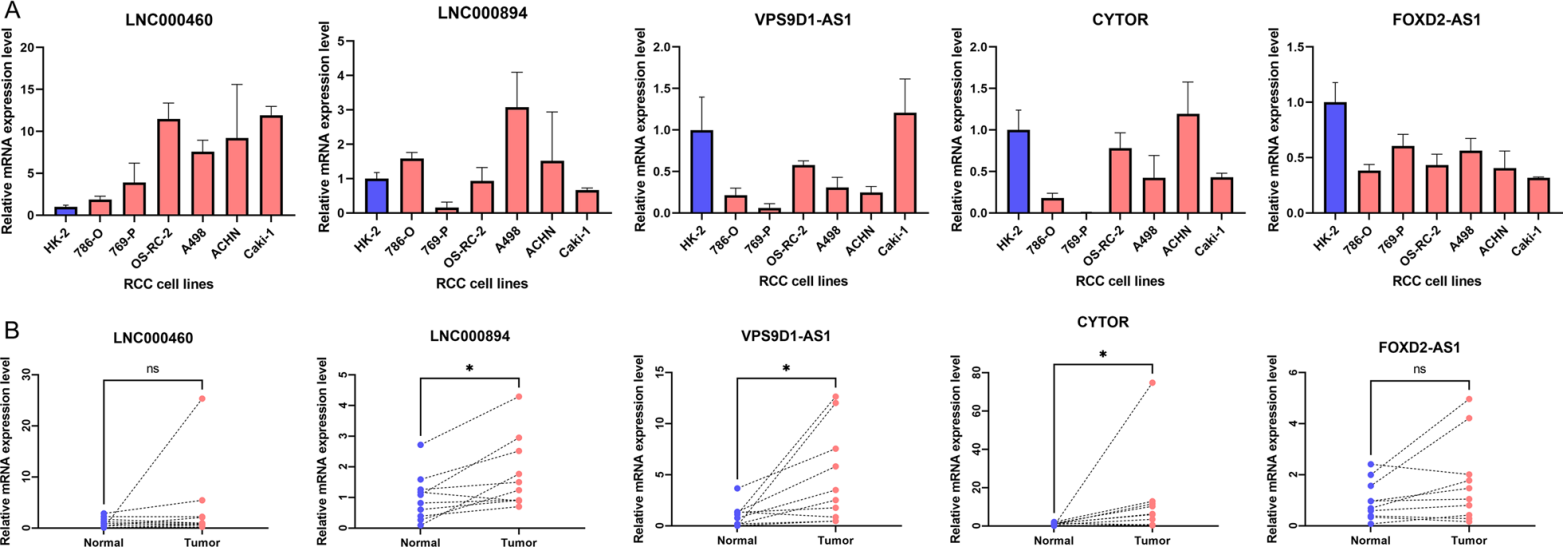
Validation of expression levels of LncRNAs in signaturein cell lines and samples. **A** Levels of mRNA expression of LncRNAs in cell lines. **B** The mRNA expression levels of LncRNAs in 10 pairs of paired clinical samples. *P < 0.05

We finally validated the 5 lncRNAs in signature in ccRCC clinical samples and RCC cell lines. Compared to the normal cell line HK-2, the expression levels of LncRNAs in RCC cell lines were inconsistent, with higher or lower levels present (Fig. 9A). The results may be due to the inability of a single cell line to mimic the overall situation of RCC and adjacent normal tissues. We then examined the expression levels of the corresponding mRNAs in 10 paired clinical samples, in which LNC000894, VPS9D-AS1, and CYTOR were highly expressed in cancer, while LNC000460 and FOXD2-AS1 were not evident (Fig. 9B).

## Discussion

The ccRCC is a molecularly heterogeneous tumor characterized as radiotherapy and chemotherapy-resistant [2, 5]. With the development of diagnostic and therapeutic techniques, the 5-year survival rate of ccRCC patients has been significantly improved. However, 25–30% of ccRCC patients have metastases at initial diagnosis [4]. The 5-year survival rate is merely 10%. Most ccRCC patients have no apparent symptoms such as pain and hematuria, usually resulting in diagnostic difficulty in the early stage [27]. Moreover, the current TNM staging system used in clinical practice lacks accuracy for prognostic evaluation [28, 29]. The early diagnosis and accurate assessment of ccRCC patients remain challenging for the reasons mentioned above. Therefore, it is still vital to screen out new clinical and molecular biomarkers for diagnosis and treatment.

Ferroptosis plays essential roles in the progression and tumorigenesis of RCC [30]. Notably, the expression levels of various ferroptosis-related genes were significantly correlated with the prognosis of ccRCC patient, suggesting that targeting ferroptosis-related pathway might be an effective option for ccRCC treatment [31, 32]. Moreover, lncRNAs plays important roles in regulating the expression of FRGs and the process of ferroptosis [33, 34]. Hence, in this study, we comprehensively analyzed the expression and prognosis of ferroptosis-related lncRNAs. And then, we performed molecularly typing and developed a prognostic model based on 5 lncRNAs in patients with ccRCC, using the method above.

Although many molecular subtypes of ccRCC based on gene expression have been proposed in recent years, the cluster of lncRNAs associated with ferroptosis has not been fully explored [35]. Therefore, we divided ccRCC patients into two clusters based on prognostic DEFRlncRNAs using the NMF algorithm. PCA analysis showed significant differences between the two clusters. The Kaplan–Meier plot showed that cluster 1 had a worse prognosis than cluster2. GSVA analysis showed that many tumor-related pathways were significantly altered between the two clusters (such as hypoxia and coagulation), suggesting that patients in the two clusters may have different sensitivities to some clinical drugs. We then tested the sensitivity of different cluster of patients to some drugs commonly used in advanced kidney cancer. The results showed promising differences, which contributed to the implementation of individualized treatment.

The immune components of the tumor microenvironment and immune cells significantly regulate tumor development [36]. We further compared the differences in immune cell infiltration between the two clusters. We found high levels of the CD8 + T cells, T cell regulatory, follicular helper T cells, and memory B cells were presented high expression levels in cluster 1. In contrast, the expression levels of neutrophils and macrophages were significantly increased in cluster 2. Unlike most cancer types, previous studies have shown that the density of CD8 T cells is correlated with poor prognosis in patients with RCC [37, 38]. This also explains why patients in cluster 1 have a worse prognosis. Moreover, the infiltration of mesenchymal cells and neutrophils may serve as a protective factor for RCC. We then examined at the mRNA expression levels of common therapeutic targets, and patients in cluster 1 had significantly upregulated expression levels of PD1, CTLA4, and other genes. We also examined IPS under different immunotherapy modalities through the TCIA database. We found higher CTLA4 and CTLA4 + PD1 IPS in cluster1, suggesting that patients in cluster1 may be more sensitive to immunotherapy. However, cluster 1 patients showed a worse prognosis in previous results. This suggests that the sensitivity of immunotherapy may not have a significant impact on prognosis.

Afterward, we established a predictive signature using LASSO regression. The Kaplan–Meier plot and time-dependent ROC curves showed that the predictive signature exhibited good predictive performance. Furthermore, most of the lncRNAs in our signature have been reported in various cancer types. LINC00460 has been extensively studied in cancer and has been shown in several studies to be a prognostic target in renal clear cell carcinoma [39–41]. LINC00894 was reported to promote breast cancer metastasis by regulating ZEB1 [42]. CYTOR and VPS9D1-AS1 are associated with the prognosis of multiple cancers and can regulate the progression of multiple cancers by sponging miRNAs [43–45]. Similarly, FOXD2-AS1 was strongly associated with the prognosis and progression of cancer patients in various cancers [46, 47]. These validation results in multiple datasets and literature mining results indicate that the prognostic signature predicts the prognosis of ccRCC patients and may function as the regulator of ccRCC progression.

Nevertheless, this study has several certain limitations. First, our data are based on the TCGA and ICGC databases, and more independent datasets are needed for testing and validation. Second, some lncRNAs in our signature play an essential role in cancer and need to be validated in future experiments. In conclusion, we first systematically analyzed the expression and prognostic value of ferroptosis-related lncRNAs and assessed immune infiltration and potential prognostic targets by molecular subtyping in ccRCC patients.

## Conclusion

In conclusion, our study identified the FRDELncRNAs and successfully constructed an individualized ccRCC signature (riskScore), which proved to be significantly correlated with OS in both the training and validation cohorts. We also estimated the potential relationships among immune cell infiltration, immunotherapy-related targets, and potential therapeutic drugs between the two molecular subtyping clusters. Our research is anticipated to provide new insights into ferroptosis-related lncRNAs for future work.

## Supplementary Information

**Additional file 1: Table S1. **Clinical information of 530 ccRCC patients.

**Additional file 2: Table S2. **Detailed information of the 10 paired clinical specimens.

**Additional file 3: Table S3. **Paired primers of the lncRNAs in the FerroSig.

**Additional file 4: Table S4. **The gene list of the ferroptosis-related genes.

**Additional file 5: Figure S1. **KM-Plot for overall survival and disease-free survival, based on 5 LncRNAs in signature.

## Data Availability

Public databases analyzed in this study could be found here: TCGA (https://portal.gdc.cancer.gov/) and ICGC database (http://daco.icgc.org/). The authors will provide the original data supporting the conclusions of this paper without undue reservation.

## Abbreviations

RCC: Renal cell carcinoma
ccRCC: Clear cell renal cell carcinoma
TNM: Tumor node metastasis
LncRNAs: Long non-coding RNAs
FRGs: Ferroptosis-related genes
TCGA: The Cancer Genome Atlas
GEO: Gene expression omnibus
DELncRNAs: Differentially expressed LncRNAs
FRDELncRNAs: Ferroptosis-related differentially expressed LncRNAs
KEGG: Kyoto encyclopedia of genes and genomes
GSVA: Gene set variation analysis
GDSC: Genomics of drug sensitivity in cancer
LASSO: Least absolute shrinkage and selection operator
ROC: Receiver operating characteristic
AUC: Area under the curve
OS: Overall survival
