# Supplementary material for: Construction and validation of a ferroptosis-related long noncoding RNA signature in clear cell renal cell carcinoma
Source: Cancer Cell Int. 2022 Sep 14;22:283. doi: 10.1186/s12935-022-02700-0 (PMC9476564; doi:10.1186/s12935-022-02700-0)
Supplement: Supplementary file 1 — Additional file 1: Table S1. Clinical information of 530 ccRCC patients. [file 12935_2022_2700_MOESM1_ESM.docx]

| **Additional file 1: Table 1 Clinical information of 530 ccRCC patients** | | | |
| --- | --- | --- | --- |
| Clinical parameters | Variable | Total (530) | Percentages (%) |
| Age | ≤60 | 263 | 49.62 |
|  | >60 | 267 | 50.38 |
| Gender | Male | 344 | 64.91 |
|  | Female | 186 | 35.09 |
| Pathological T | T1 | 271 | 51.13 |
|  | T2 | 69 | 13.02 |
|  | T3 | 179 | 33.77 |
|  | T4 | 11 | 2.08 |
| Pathological M | M0 | 420 | 79.25 |
|  | M1 | 78 | 14.72 |
|  | MX | 32 | 6.04 |
| Pathological N | N0 | 239 | 45.09 |
|  | N1 | 16 | 3.02 |
|  | NX | 275 | 51.89 |
| AJCC stage | Stage I | 265 | 50.00 |
|  | Stage II | 57 | 10.75 |
|  | Stage III | 123 | 23.21 |
|  | Stage IV | 83 | 15.66 |
|  | Unkonwn | 2 | 0.38 |
| ISUP grade | G1 | 14 | 2.64 |
|  | G2 | 227 | 42.83 |
|  | G3 | 206 | 38.87 |
|  | G4 | 75 | 14.15 |
|  | GX | 8 | 1.51 |
| Survival status | Dead | 173 | 32.64 |
|  | Alive | 357 | 67.36 |
