# Supplementary figures and images for: Construction and validation of a ferroptosis-related long noncoding RNA signature in clear cell renal cell carcinoma
Source: Cancer Cell Int. 2022 Sep 14;22:283. doi: 10.1186/s12935-022-02700-0 (PMC9476564; doi:10.1186/s12935-022-02700-0)

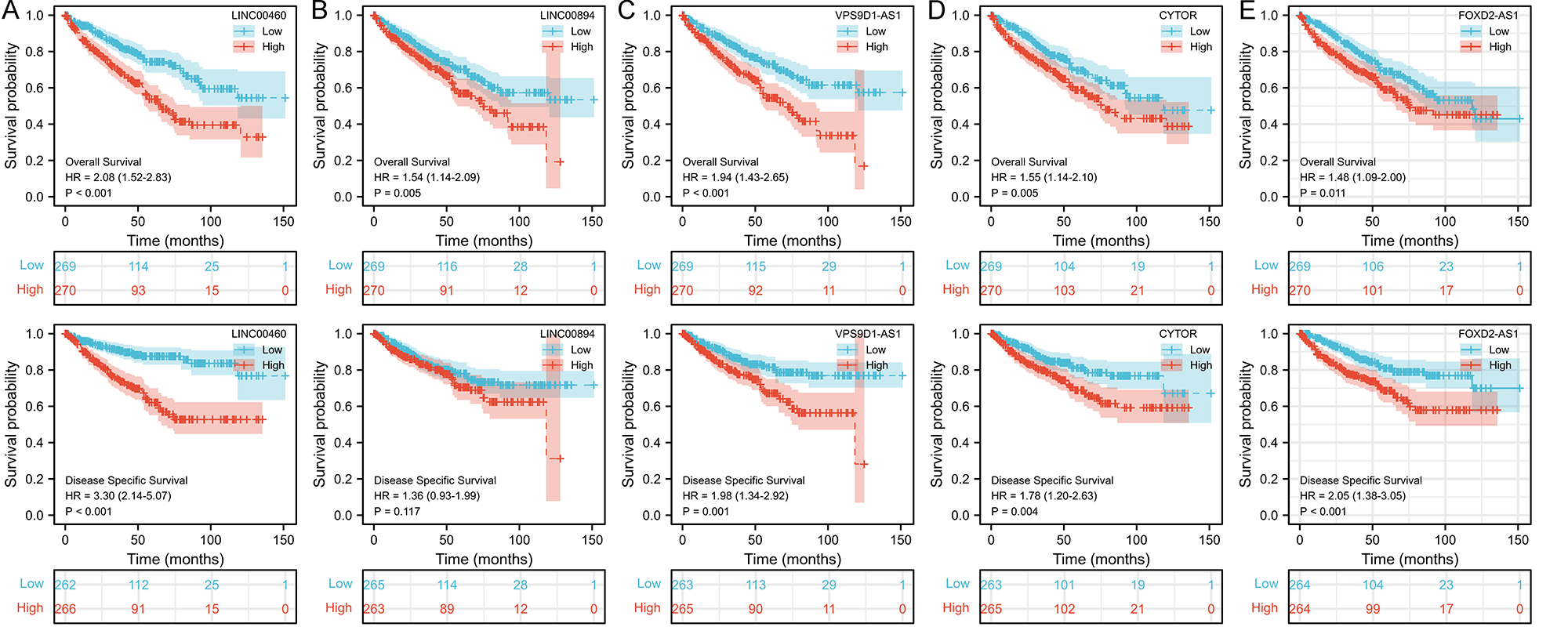

Supplement: Supplementary file 5 — Additional file 5: Figure S1. KM-Plot for overall survival and disease-free survival, based on 5 LncRNAs in signature. [file 12935_2022_2700_MOESM5_ESM.tif]
